# Response: Commentary: The effects of postponing BCG vaccination on the risk of BCG-related complications among patients with severe combined immunodeficiency disease in Saudi Arabia

**DOI:** 10.3389/fimmu.2026.1872494

**Published:** 2026-07-09

**Authors:** Hamoud Al-Mousa, Suliman Aljumaah

**Affiliations:** 1 Section of Pediatric Allergy and Immunology, Department of Pediatrics, King Faisal Specialist Hospital & Research Center, Riyadh, Saudi Arabia; 2 Section of Infectious Diseases, Department of Pediatrics, King Faisal Specialist Hospital & Research Center, Riyadh, Saudi Arabia

**Keywords:** Bacillus Calmette Guerin, BCG, Saudi Arabia, severe combined immunodeficiency disease, tuberculosis, vaccination

We thank the authors for their commentary on our article and for raising public health considerations regarding Bacillus Calmette–Guérin (BCG) vaccination policy. We would like to reiterate that our study was designed with a clearly defined and focused objective: to evaluate the impact of postponing BCG vaccination on BCG-related complications among patients with severe combined immunodeficiency (SCID). Within this scope, we demonstrated a marked and clinically meaningful reduction in BCG-related complications from 46.1% to 2.6% following the policy change. These findings are robust, biologically plausible, and directly relevant to this high-risk population. Accordingly, interpretation of our results should remain aligned with this objective, and any argument opposing these conclusions should be supported by direct evidence demonstrating harm in SCID patients rather than theoretical extrapolation to the general population.

A central concern raised in the commentary is that delaying BCG vaccination may increase tuberculosis (TB) burden in the general population. However, it is important to emphasize a fundamental point: BCG vaccination does not reliably prevent primary infection with *Mycobacterium tuberculosis*, nor does it prevent reactivation of latent pulmonary TB, which remains the principal driver of transmission within the community. The protective effect of BCG is largely confined to severe manifestations such as TB meningitis and disseminated TB (1, 2). Consequently, the assumption that delaying BCG vaccination would significantly alter community transmission dynamics is not supported by established biological or epidemiological evidence. Importantly, real-world data from the Saudi Ministry of Health demonstrate that TB incidence has continued to decline following the implementation of the 2019 policy, with no signal suggesting an increase in TB burden with the most recent reported incidence being 8.4 per 100,000 (3–5). This directly contradicts the assertion that postponing BCG vaccination to 6 months has adversely impacted TB control. In this context, any position opposing the current policy should be substantiated by robust epidemiological data demonstrating increased TB incidence or severe pediatric TB outcomes, which is currently lacking.

Vaccination strategies must be contextualized to local epidemiology and population-specific risks. In Saudi Arabia, the high rate of consanguinity has resulted in a significantly increased prevalence of inborn errors of immunity. We have previously shown that the incidence of SCID in our population is approximately 1 in 2,906 live births, one of the highest reported worldwide (6). This epidemiological reality translates into a substantially higher risk of severe, disseminated, and often fatal BCG-related complications compared to populations with lower rates of immunodeficiency. Indeed, prior to the policy change, BCG-related disease represented a significant and preventable contributor to morbidity and mortality in this group. Therefore, vaccination policy in our setting must carefully balance population-level benefits against the risk of catastrophic outcomes in a vulnerable subgroup. In such a context, even a relatively small proportion of affected infants represents a significant clinical and ethical burden that must be addressed.

We fully agree with the authors that newborn screening using T-cell receptor excision circle (TREC)-based assays represents the optimal strategy for early identification of SCID and safe implementation of BCG vaccination. Indeed, we have previously demonstrated the need for such screening in Saudi Arabia and have strongly advocated for its national implementation. However, this infrastructure is not yet established in the kingdom. In the absence of newborn screening, delaying BCG vaccination to 6 months represents a pragmatic and evidence-based interim strategy that allows time for clinical recognition of immunodeficiency and thereby reduces the risk of severe vaccine-related complications. The alternative 28-day vaccination strategy proposed in the commentary is applicable primarily in settings where newborn screening is already in place and SCID can be reliably excluded prior to vaccination. Without such screening, a 28-day delay does not adequately mitigate risk, as SCID diagnosis is frequently delayed beyond the neonatal period. Therefore, the current Saudi approach reflects a context-specific public health decision tailored to existing healthcare infrastructure rather than a simplified binary comparison.

We also acknowledge the importance of broader public health considerations raised in the commentary, including vaccine coverage, defaulter rates, and access barriers among vulnerable populations. These are valid concerns that warrant careful evaluation through national immunization registries and population-based studies. However, these issues remain largely theoretical in the absence of systematic data demonstrating a measurable adverse impact following the policy change. At present, available national data do not indicate an increase in TB incidence, severe pediatric TB, or TB-related mortality following the implementation of the delayed vaccination policy. We fully support the call for prospective national studies to address these gaps and strengthen surveillance systems.

It is also important to highlight that World Health Organization recommendations regarding BCG vaccination are based on the underlying epidemiology of TB within each country (7). In settings with low TB incidence, several countries in Europe and North America have adopted selective vaccination strategies, reserving BCG for high-risk populations rather than implementing universal neonatal vaccination. Current epidemiological data indicate that Saudi Arabia falls within a relatively low TB incidence category, with a continued declining trend. This raises a broader and legitimate question as to whether universal BCG vaccination is required for the entire population or whether a more targeted, risk-adapted strategy may be appropriate in the future. While this question extends beyond the scope of our study, it further underscores the importance of tailoring vaccination policies to national epidemiology rather than applying uniform global assumptions.

Finally, while emerging data on heterologous or “trained immunity” effects of neonatal BCG are acknowledged, these potential benefits must be interpreted within context and balanced against the well-established and immediate risk of severe, disseminated, and potentially fatal BCG-related complications in immunocompromised infants. Similarly, while substrain variability may influence the frequency of adverse events, it does not negate the fundamental role of host immune status and timing of vaccination in determining risk. The observed reduction in complications following delayed vaccination is consistent with established pathophysiological understanding and reinforces the importance of timing as a modifiable risk factor.

In conclusion, we agree that a scientifically rigorous policy evaluation should consider both benefits and potential risks at the population level, and we strongly support the need for prospective national studies evaluating TB outcomes, vaccination coverage, and access disparities. We also reaffirm that implementation of newborn screening for SCID represents the optimal long-term strategy. However, based on currently available evidence, delaying BCG vaccination to 6 months has resulted in a substantial reduction in life-threatening complications in SCID patients, without evidence of increased TB burden at the population level. In a setting characterized by a high prevalence of inborn errors of immunity and the absence of newborn screening, this approach represents a pragmatic, context-appropriate, and evidence-based strategy to improve patient safety while broader public health measures continue to evolve.
